# Novel Cemented Carbide Inserts for Metal Grooving Applications

**DOI:** 10.3390/ma18153674

**Published:** 2025-08-05

**Authors:** Janusz Konstanty, Albir Layyous, Łukasz Furtak

**Affiliations:** 1 Faculty of Metals Engineering and Industrial Computer Science, AGH University of Krakow, 30 Mickiewicz Avenue, 30-059 Krakow, Poland; 2 P.H.M. POLCOMM, Chlewiska 100, 21-100 Lubartów, Poland

**Keywords:** powder metallurgy, cemented carbides, grooving inserts

## Abstract

Although cemented carbides have been manufactured by the powder metallurgy (P/M) technology for over a century now, systematic developmental efforts are still underway. In the present study, tool life improvements in metal grooving applications are the key objective. Four PVD-coated cemented carbides compositions, dedicated to groove steel, stainless steel, cast iron, and aluminium alloys, have been newly designed, along with their manufacturing conditions. Physical, mechanical and chemical characteristics—such as sintered density, modulus of elasticity, hardness, fracture toughness, WC grain size, and the chemical composition of the substrate material, as well as the chemical composition, microhardness, structure, and thickness of the coatings—have been studied. A series of grooving tests have also been conducted to assess whether modifications to the thus far marketed tool materials, tool geometries, and coatings can improve cutting performance. In order to compare the laboratory and application properties of the investigated materials with currently produced by reputable companies, commercial inserts have also been tested. The experimental results obtained indicate that the newly developed grooving inserts exhibit excellent microstructural characteristics, high hardness, fracture toughness, and wear resistance and that they show slightly longer tool life compared to the commercial ones.

## 1. Introduction

Cemented carbides, also termed as hardmetals, belong to a group of hard and wear-resistant materials in which the hard carbide particles (WC, TiC, TaC, NbC, etc.) are cemented by means of a ductile cobalt or, less frequently, a nickel binder. Cemented carbides are fabricated exclusively by the P/M route using various powder shaping and sintering techniques [1]. For metal cutting applications, the tool insert performance strongly depends on the type and amount of the carbide phase and its grain size, the amount of binder metal, and, more importantly, the presence, or absence, of thin hard coatings deposited on the working surface of the tool [2]. In the vast majority of applications, straight WC-Co grades are used, with WC grain size typically ranging between 0.5 and 2.0 μm [1]. The tool material is most often coated to provide protection against extreme cutting conditions such as those characterised by high temperature, oxidation, corrosion, friction, and wear [3]. Useful information on different kinds of coatings with respect to their deposition technology [4], internal structure [5,6,7,8,9,10], hardness [4,9], thermal conductivity [11,12], adhesion to the substrate [13,14], effects on cutting forces [15], tool life [10,15,16,17,18,19,20,21] and wear behaviour [22,23,24], resistance to corrosion [25,26], and quality of surface machining [27] is readily available in the literature.

A literature review suggests that improvements in tool life have mostly been sought through engineering coatings with a nano-structured [13,17] multilayered architecture [4,5,6] to achieve high-strength adhesion with the tool substrate [7,13,14] and ensure low adhesion of the workpiece material to the cutting edge surface [15,23], designed to easily remove heat from the cutting region [11,16,24]. It is believed that the commonly used PVD magnetron sputtering techniques provide the best combination of enhanced adhesion strength and uniform film growth [25].

WC-Co tools have long been used successfully for cutting cast irons and nonferrous metals. They have been less successful, however, in cutting steels. In this case, the wear, based on diffusion, leads to rapid cratering, which is amplified by cutting speed. Therefore the tool fails at speeds only slightly exceeding those used with high-speed steels. One possible solution to this problem is to use WC-TiC-Co or WC-TiC-TaC-Co grades, which exhibit higher hardness and compressive strength at high temperatures, combined with resistance to crater formation [28,29]. Application of protective coatings is another solution [29]. Monolayer or multilayer coatings are applied to steel cutting tools in order to give cutting edge characteristics that are quite different from those of the body of the insert. Coatings of various kinds, including titanium, hafnium, tantalum, and zirconium carbides and nitrides; alumina/titanium oxide combinations; and multiple carbide/carbonitride/nitride/oxide, oxynitride, or oxycarbonitride combinations, have been recently developed and used with great success [2,30]. At present, uncoated cemented carbide grades represent only a very small portion of the total cutting tool assortment.

The main objective of the present work was to develop novel cemented carbide inserts for grooving metals and their alloys, along with the technology needed for their industrial production. The new tools would include four grades of PVD-coated sintered carbides dedicated to groove steel (ISO group P), stainless steel (ISO group M), cast iron (ISO group K), and nonferrous alloys (ISO group N). The research was technological in nature and aimed to create products that are competitive or superior to those produced by reputable companies. The novelty of this research involved the development of new substrate/coating material combinations and the refinement of technological parameters for their production

## 2. Materials and Methods

Four proprietary compositions of WC-Co base cemented carbides were selected for this work. The experimental materials were formulated to achieve the properties presented in Table 1.

**Figure 1:**
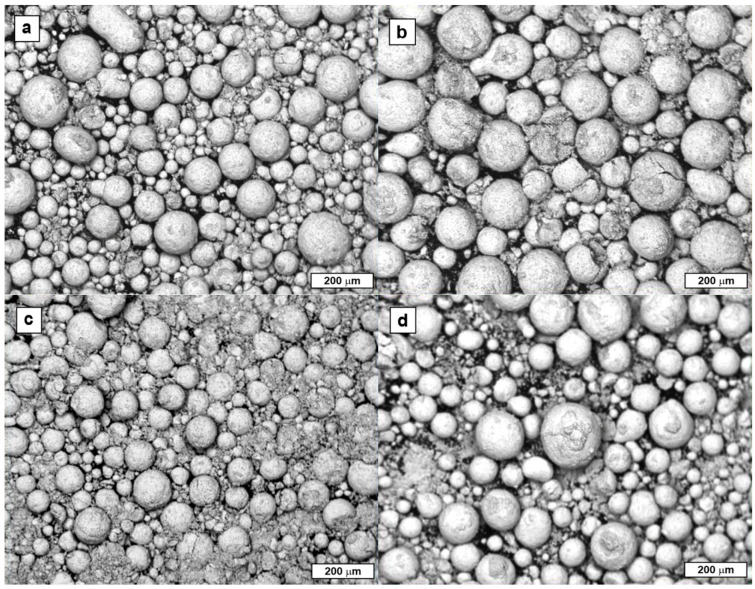
Micrographs of granulated powders dedicated to fabrication of (**a**) P, (**b**) M, (**c**) K, and (**d**) N group inserts.

The starting powders that met the assumed chemical compositions and WC grain sizes were delivered in the granulated condition, as shown in Figure 1, by a commissioned manufacturer.

They were used to prepare the experimental samples by the P/M press and sinter-HIP route. After preliminary verification of the powder consolidation conditions with respect to phase composition, WC grain size, sintered hardness, and porosity, the pressing parameters and sintering curves were duly corrected/optimised. Afterward, approximately 1000 double-ended inserts of each composition were manufactured. The sintered pieces were again tested for WC grain size, fracture toughness, hardness, density, porosity, magnetic coercivity, and saturation using Polcomm’s quality control methodologies in order to reject inserts that did not meet the material and geometrical assumptions. Monolayer or multilayer coatings were subsequently deposited on the majority of the cemented carbide inserts via the PVD High-Power Impulse Magnetron Sputtering (HIPIMS) technique.

Both uncoated and coated grooving inserts were subjected to laboratory tests. Selected samples were tested for hardness, fracture toughness, and tribological properties and subjected to microstructural studies.

The Vickers method was used to test hardness. The measurements were carried out using the FLC-50VX hardness tester (Future-Tech, Kawasaki, Japan) in compliance with PN EN 843-4:2007 [31]. The average of twenty readings was reported as the Vickers hardness of the material.

Fracture toughness was determined by the critical stress intensity factor (*K*_Ic_), measured by the indentation method using the Palmqvist corner cracks model proposed by Niihara et al. [30]. The critical stress intensity factor was the average of readings taken around twenty Vickers diamond indentations made at 30 kgf. The following equation for the critical stress intensity factor was used [30]:

(1) $$K_{Ic}=\frac{0.043\cdot H\cdot a^{\frac{3}{2}}}{{\left(\frac{H}{3E}\right)}^{\frac{2}{5}}\cdot c\sqrt{\frac{c}{a}}}$$

where *H* is the hardness (HV30), *E* is the Young’s modulus, *a* is the half-diagonal of the Vickers indentation, and *c* is the distance from the centre of indentation to the crack tip.

From Equation (1), it can be seen that *K*_Ic_ is related to Young’s modulus. Therefore, measurements of *E* were performed by the ultrasonic method prior to toughness tests. The EPOCH-3 ultrasonic flaw detector (Panametrics, Inc., Waltham, MA, USA), equipped with broadband ultrasound generating heads, was used in these measurements. The following formula was used to determine the Young’s modulus [32]:

(2) $$E=\rho V_{T}^{2}\frac{3V_{L}^{2}-4V_{T}^{2}}{V_{L}^{2}-V_{T}^{2}}$$

where *ρ* is the density of the tested material; *V*_T_ and *V*_L_ are the velocities of the transverse and longitudinal waves.

In order to provide data for Equation (2) but also ensure that sufficient densification had been achieved, the vast majority of inserts were tested for sintered density. A method based on the Archimedes’ principle was carried out according to PN-EN ISO 18754:2022 [33].

The wear tests were carried out on both uncoated and PVD-coated samples. The pin-on-disc CETR-UMT-2MT tribometer (Brucker, Billerica, MA, USA) was used to measure the friction coefficient and wear in dry contact conditions. Quenched and tempered 41Cr4 steel, X5CrNiMo17-12-2 stainless steel, GJS-400-15 nodular cast iron, and AW-2017A aluminium alloy were used as rotating discs for pins made out of P, M, K, and N grooving inserts, respectively. The stationary pins (grooving inserts) were pressed against the rotating discs under 10 N load. The wear track diameter, sliding distance, and velocity were 0.3 m, 1000 m, and 0.2 m/s, respectively.

The friction force was continuously monitored throughout the test. After running-in, when the system attained the steady-state condition, the average value of friction force was estimated and used to calculate the coefficient of friction (COF). After completion of the test, the maximum width of flank surface wear of each tested insert was measured to give the estimate of wear resistance.

Microstructural analyses and imaging were performed using the ECLIPSE LV150N metallographic light microscope (LM) (Nikon, Tokyo, Japan) and the JSM-6460LV JSM-6460LV scanning electron microscope (SEM) (Jeol, Tokyo, Japan). The SEM was equipped with an energy-dispersive X-ray spectrometer (EDS) (Oxford Instruments, Abingdon, UK), which allowed for qualitative and quantitative elemental analysis in microareas.

The dimensions of WC grains were estimated on metallographic cross-sections [34]. The intercept method, ideally suited for measuring nonequiaxed grains, was chosen to determine the planar WC grain size. The mean intercept length (mean WC grain size) was calculated as follows:

(3) $${\bar{L}}_{3}=A_{A}/N_{L}$$

where *A*_A_ is the areal fraction of WC particles, and *N*_L_ is the number of intersections with WC particles by the straight test line of length *L*.

Characterisation of porosity and detection of other defects such as graphite or eta-phase inclusions were also carried out on metallographic cross-sections etched in Murakami’s reagent. Pores sizes were assessed at a magnification of ×200 and compared with the range of photomicrographs contained in ISO 4499-4:2016 [35]. The porosity levels were reported by reference to the appropriate photomicrograph and designated using the A and B scales. Uncombined carbon was assessed in a similar way and reported using the C scale.

**Figure 2:**
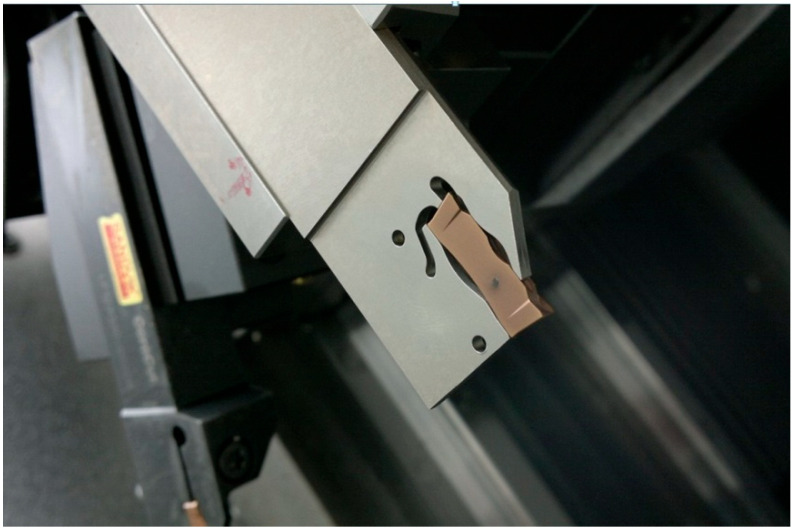
Grooving insert clamped in a double-ended insert mounting blade.

Cutting tests were carried out using the CNC DMG MORI NEF 400 universal turning machine (Mach4Metal BV, Babberich, Holand) powered by a 18.5 kW main motor. Additionally, 3 mm wide double-edge grooving inserts were mounted laterally in a specially designed Polcomm DEBL 32N30 tool holder, shown in Figure 2.

The main cutting test conditions are listed in Table 2.

During grooving tests, the flank wear of the straight part (zone B) of the cutting edge (*VB*_B_) was used as the main tool life criterion. The length of time needed to develop the average width of the regularly (uniformly) worn flank wear land *VB*_B_ = 0.3 mm was recorded and taken as the measure of tool life according to ISO 3685:1993 [36].

Simultaneously with the developmental work, carbide grooving inserts produced by a number of reputable companies were also tested under the same conditions in order to benchmark the newly developed materials against the commercial ones.

## 3. Results and Discussion

### 3.1. Laboratory Tests

Prior to coating, cemented carbide inserts from all of the investigated application groups were tested for sintered density, modulus of elasticity, hardness and fracture toughness. The latter two properties were measured on metallographic cross-sections. The average results are summarised in Table 3.

The tests presented above were complemented with metallographic analysis to identify and quantitatively characterise microstructural features such as average WC grain size, A- and B-type porosity, and presence of inclusions of uncombined carbon or eta-phase.

**Figure 3:**
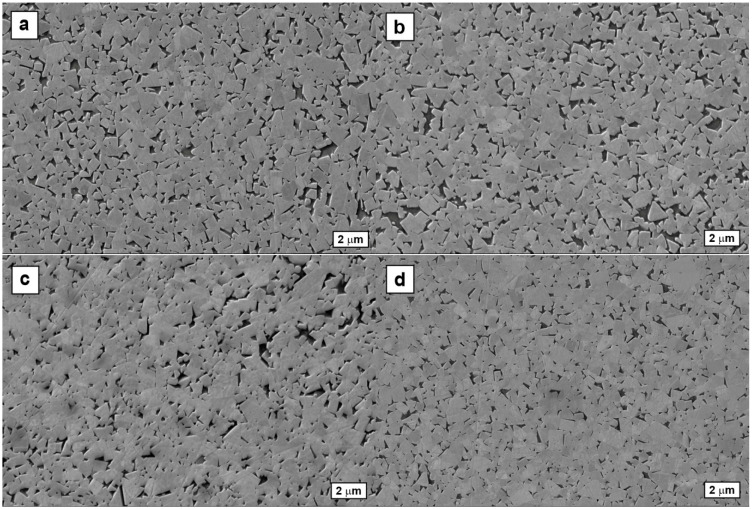
SEM-SE micrographs showing typical microstructures of cemented carbide inserts that belong to different ISO application groups: (**a**) P, (**b**) M, (**c**) K, and (**d**) N.

The results of the metallographic studies are presented in Figure 3 and in Table 4.

The metallographic cross-sections were also used to assess chemical compositions of inserts using energy-dispersive X-ray spectrometry (EDS). The results are presented in Table 5.

The experimental results show that the commissioned WC–base powders can readily be consolidated by the cold press/sinter–HIP route to satisfy the basic compositional and microstructural requirements given in Table 1. From Table 5, it can be seen that all materials contain small additions of chromium carbide that are sufficient to prevent WC grain growth, increase mechanical properties, and lower the sintering temperature [37].

The sintered densities are near-theoretical (see Figure 3 and Table 4), i.e., neither A- and B-type porosity nor C-type uncombined carbon was detected as per ISO 4499-4:2016 [35]. Similarly, the eta-phase that forms in carbon-deficient materials was not found in the examined samples.

Table 3 shows the comparisons between the achieved hardness and fracture toughness values and those characteristic for commercial materials. It is clear from these results that the mechanical characteristics of the experimental materials are comparable to those of cemented carbides produced on a commercial scale.

At this stage of the R&D agenda, the acceptance criteria for all four grades of cemented carbides were met. Therefore, the majority of the grooving inserts were subjected to deposition of coatings by the PVD HIPIMS technique. To avoid coating delamination, i.e., to create strong bonding between the cemented carbide substrate and the coating, thin titanium nitride interlayers were deposited.

The assumed coating characteristics and deposition conditions are summarised in Table 6.

After deposition, the coatings were tested for Vickers microhardness, thickness, and chemical composition. The microhardness readings were taken on both metallographic cross-sections and outer surfaces. The coating thickness was measured microscopically at minimum five random points on both flank and rake surfaces. The chemical compositions of the coatings were roughly estimated by SEM-EDS within selected areas.

The average coating microhardness and total thickness values are summarised in Table 7.

**Figure 4:**
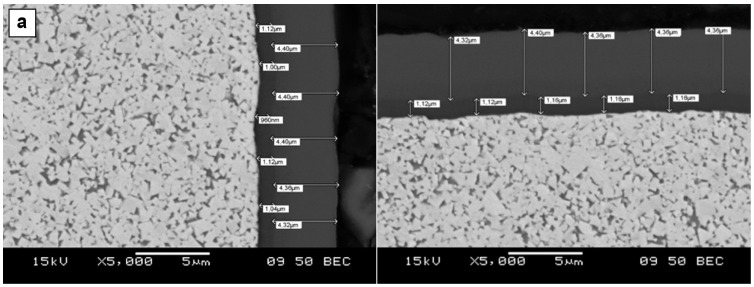

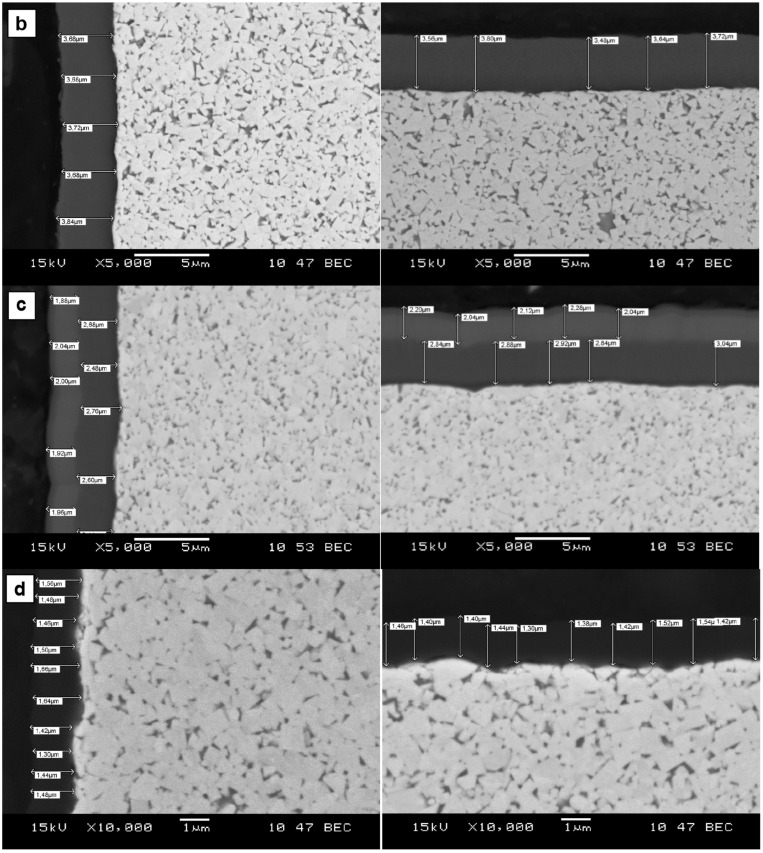
Micrographs showing thickness of coatings deposited on flank faces (left) and rake faces (right) of (**a**) P, (**b**) M, (**c**) K, and (**d**) N group inserts.

The individual coating thickness measurements are presented in Figure 4.

The coating composition estimates are given in Table 8.

It is evident from the results shown in Figure 4 and Table 7 and Table 8 that the targeted coating characteristics given in Table 6 were achieved. The lower values of the obtained hardness numbers are due to the excessively high ratio of indentation diagonal length to coating thickness. For this reason, the hardness of the DLC coating was only measured on its outer surface. Minor deviations from the assumed chemical compositions presumably originated from interdiffusion of elements between the substrate and the coating layers at the coating deposition step.

**Figure 5:**
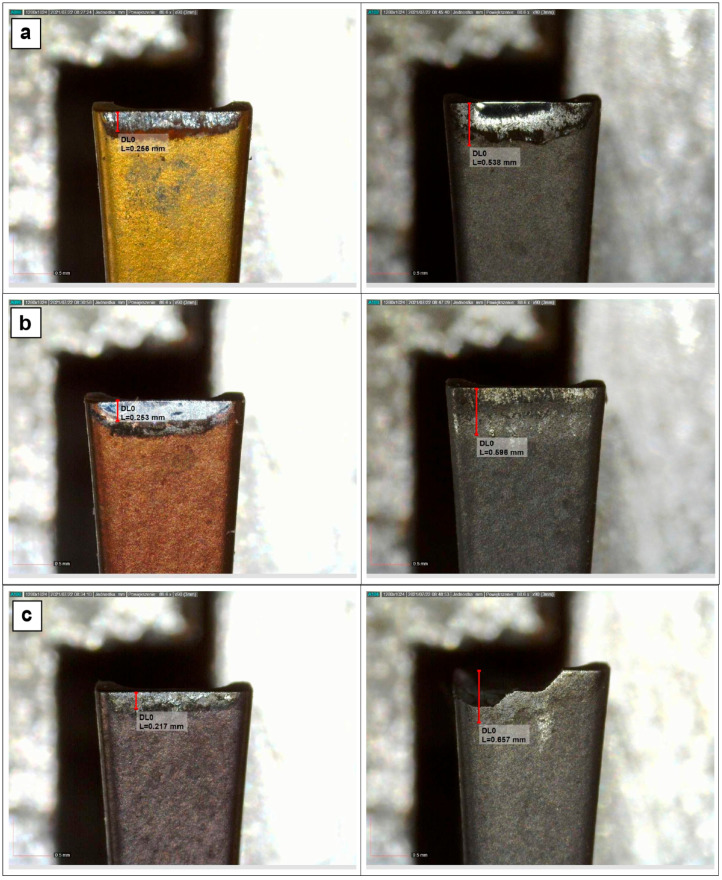

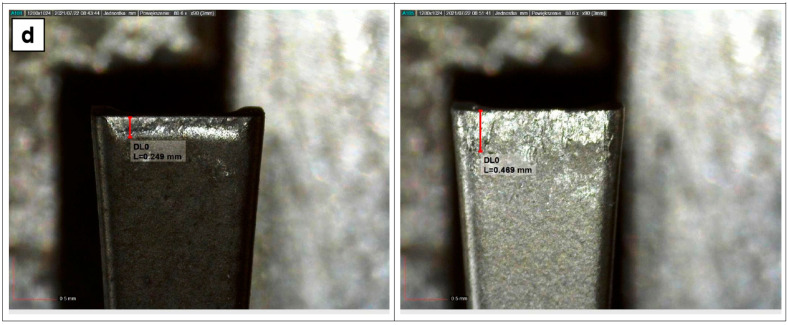
Flank wear lands developed on coated (left) and uncoated (right) inserts after sliding for a distance of 1000 m: (**a**) P, (**b**) M, (**c**) K, and (**d**) N group inserts.

Because the acceptance criteria for all four types of coatings were also met, the coated and uncoated grooving inserts were subjected to the pin-on-disc wear test described in Section 2. The widths of the flank wear lands were measured and used as wear resistance estimates, as exemplified in Figure 5.

The pin-on-disc wear test results are summarised in Table 9.

The wear test results show that deposition of coatings on the cemented carbide grooving inserts has a negligible effect on the coefficient of friction but leads to marked improvements in wear resistance. The effect of the coefficient of friction on the rate of wear seems to be a complex issue to which there is no straightforward answer.

### 3.2. Grooving Tests

The metal grooving tests were performed with 3 mm wide double-edge grooving inserts installed in the Polcomm DEBL 32N30 highly rigid self-clamping mounting blade shown in Figure 2. The tool holder geometry and dimensions of all tooling members are given in Ref. [38].

The main cutting test conditions and tool life data are listed in Table 10.

The grooving test results indicate that the newly developed, PVD-coated cemented carbide inserts perform at least similarly to, or better than, the competitive commercial tools. When the flank wear land *VB*_B_ = 0.3 mm is chosen as the tool failure criterion, the tested inserts show stable and predictable tool life with very little variation between individual measurements. From Table 5, it is clear that the tool materials developed within this project can compete with the materials available on the market in terms of cutting performance.

## 4. Conclusions

Four industrially promising WC-Co-based cemented carbides with minor additions of Cr_3_C_2_ and, in one case, (Ta,Nb)C were produced in the form of grooving inserts with either monolayer or multilayer PVD coatings deposited on their working surfaces. Their physical, mechanical, and chemical properties (sintered density, Young’s modulus, Vickers hardness, fracture toughness, wear resistance, and compositions of the carbide substrate and coating), as well as their microstructural characteristics (WC grain size, residual porosity, and absence of undesirable phases), were carefully designed and studied extensively in order to satisfy stringent manufacturing process standards and application acceptance criteria.

The following conclusions are drawn from the study:

- Under the manufacturing conditions employed for the cemented carbide substrates, neither A- nor B-type porosity was detected, and similarly, neither uncombined carbon nor the eta-phase was found in the examined samples.
- The mechanical characteristics of the experimental cemented carbides, i.e., their hardness and fracture toughness, are comparable to those produced on a commercial scale by the leading tool manufacturers.
- The deposition of coatings on the cemented carbide grooving inserts had a negligible effect on the coefficient of friction but led to marked improvements in wear resistance. The decrease in wear rate was sensitive to the friction pair configuration ranging from 47 to 67% for the DLC-coated pin/Al alloy disc and AlTiN + TiB_2_-coated pin/cast iron disc, respectively.
- The grooving tests showed the superiority of the newly developed inserts compared to the other tested tools. They consistently outperformed their high-quality commercial counterparts in terms of tool life.

## Figures and Tables

**Table 1:** Basic characteristics of the experimental grooving inserts.

Material Designation (ISO Group)	Chemical Composition ^(a)^, wt.%				WC Grain Size, μm	PVD Coating
	**Co**	**(Ta,Nb)C**	**Cr_3_C_2_**	**VC**		
P	9.6–10.2	-	0.5–0.8	0.0–0.1	0.7–1.0	AlTiN + AlCrN
M	12.0–12.7	1.2–1.6	-	-	1.0–3.0	TiAlSiN
K	5.8–6.5	-	0.5–0.8	0.0–0.1	1.0–4.0	AlTiN + TiB_2_
N	4.8–5.3	-	0.2–0.3	0.0–0.1	1.0–3.0	DLC

^(a)^ balance WC.

**Table 2:** Grooving test parameters and workpiece materials.

Grooving Insert (ISO Group)	Machining Parameters		Workpiece		
	Cutting Speed, m/min	Feed Rate, mm/rev	Material	Diameter, mm	Hardness, HB
P	120	0.10	C45 steel	98	225
M	100	0.10	X5CrNi18-10 stainless steel	98	215
K	140	0.10	GJL-250 cast iron	98	250
N	300	0.08	AW-7075 aluminium alloy	98	150

**Table 3:** Physical and mechanical properties of the researched materials ^(a)^.

Grooving Inserts (ISO Group)	Density, g/cm^3^	*E*, GPa	HV30 ^(b)^	HV1	*K*_Ic_ ^(b)^, MPa∙m^1/2^
P	14.42 ± 0.03	582 ± 6	1567 ± 10 (1500–1620)	1630 ± 18	16.4 ± 0.2 (15.5–17.0)
M	14.22 ± 0.01	559 ± 4	1352 ± 7 (1290–1370)	1438 ± 22	18.6 ± 0.6 (18.0–19.0)
K	14.90 ± 0.02	619 ± 5	1561 ± 14 (1525–1615)	1650 ± 53	13.7 ± 0.3 (13.0–14.0)
N	14.90 ± 0.01	624 ± 2	1728 ± 12 (1650–1740)	1829 ± 20	11.6 ± 0.3 (11.0–12.0)

^(a)^ throughout the article, the scatter bands are estimated at a 90% confidence level. ^(b)^ values in brackets refer to properties of commercial materials.

**Table 4:** Main microstructural characteristics of the researched materials.

Grooving Inserts (ISO Group)	WC Grain Size (*L̅_3_*), μm	Pores ^(a)^		Uncombined Carbon ^(a)^ (Graphite)
		**≤10 μm**	**10–25 μm**	
P	0.9 ± 0.1	A00	B00	C00
M	2.0 ± 0.2	A00	B00	C00
K	2.4 ± 0.2	A00	B00	C00
N	2.0 ± 0.2	A00	B00	C00

^(a)^ reported in compliance with ISO 4499-4:2016 [35]; eta-phase was not detected.

**Table 5:** Chemical composition of the experimental grooving inserts.

Material Designation (ISO Group)	Chemical Composition Estimates (EDS), wt.%			
	Co	(Ta,Nb)C	Cr_3_C_2_	WC
P	10.28 ± 0.35	-	0.55 ± 0.30	bal
M	10.30 ± 0.33	1.35 ± 0.24	0.48 ± 0.30	bal
K	5.50 ± 0.61	-	0.60 ± 0.19	bal
N	4.98 ± 0.35	-	0.18 ± 0.12	bal

**Table 6:** Coating characteristics and deposition conditions.

Material Designation (ISO Group)	Coating Composition	Adhesive Layer	Total Thickness (Assumed), μm	μHV0.025 (Assumed)	Coating Deposition	
					Targets	Temperature Range, °C
P	AlTiN + AlCrN	TiN	5.0	3200	Ti Al60Ti40 Al70Cr30	450–550
M	TiAlSiN	TiN	4.0	3500	Ti Al60Ti40 TiSi34	450–550
K	AlTiN + TiB_2_	TiN	4.5	3550	Ti Al60Ti40 TiB2	450–550
N	DLC	TiN	1.5	5500	Ti C	180–200

**Table 7:** Main coating characteristics.

Material Designation (ISO Group)	Coating Composition	Total Thickness, μm	μHV0.025	
			Cross-Section	Outer Surface
P	AlTiN + AlCrN	5.46 ± 0.13	3025 ± 303	3123 ± 293
M	TiAlSiN	3.68 ± 0.19	3350 ± 240	3376 ± 218
K	AlTiN + TiB_2_	4.81 ± 0.47	3421 ± 196	3526 ± 230
N	DLC	1.46 ± 0.16	-	5389 ± 201

**Table 8:** Coating structures and chemical compositions.

Material Designation (ISO Group)	Coating Composition	
	Inner Layer ^(a)^	Outer Layer ^(a)^
P	Al_0.53_Ti_0.47_N (20%)	Al_0.59_Cr_0.41_N (80%)
M	Ti_0.63_Al_0.3_ Si_0.07_N	
K	Al_0.58_Ti_0.42_N (57%)	Ti_0.91_Al_0.09_B_2_ (43%)
N	100%C	

^(a)^ values in brackets denote fraction of the total coating thickness.

**Table 9:** Coefficients of friction and flank wear land widths.

Material Designation (ISO Group)	Coating Composition	Coefficient of Friction	Wear Land Width, μm	Decrease in Wear Rate Due to Coating
P	AlTiN + AlCrN	0.54	256	52.4%
	uncoated	0.52	538	
M	TiAlSiN	0.71	253	57.6%
	uncoated	0.77	596	
K	AlTiN + TiB_2_	0.31	217	67.0%
	uncoated	0.31	657	
N	DLC	0.40	249	46.9%
	uncoated	0.47	469	

**Table 10:** Grooving test results.

Grooving Insert	Workpiece	Cutting Speed (*Vc*) Feed Rate (*f*)	Flank Wear (*VB*_B_), mm	Working Time, min	Time to *VB*_B_ = 0.3 mm ^(a)^, min
P/AlTiN + AlCrN	C45 steel	*Vc* = 120 m/min *f* = 0.1 mm/rev	0.310	18.7	18.1 ± 0.1 (17.5)
			0.293	17.7	
			0.312	18.9	
			0.306	18.5	
M/TiAlSiN	X5CrNi18-10 stainless steel	*Vc* = 100 m/min *f* = 0.1 mm/rev	0.310	18.4	18.0 ± 0.4 (17.5)
			0.293	17.8	
			0.312	18.6	
			0.318	19.1	
K/AlTiN + TiB_2_	GJL-250 cast iron	*Vc* = 140 m/min *f* = 0.1 mm/rev	0.303	18.1	17.9 ± 0.2 (17.5)
			0.321	19.2	
			0.315	18.7	
			0.298	17.9	
N/DLC	AW-7075 aluminium alloy	*Vc* = 300 m/min *f* = 0.08 mm/rev	0.314	18.8	18.1 ± 0.4 (17.5)
			0.286	17.5	
			0.306	18.4	
			0.312	18.7	

^(a)^ values in brackets denote market competitiveness thresholds evaluated on commercial inserts.

## Data Availability

The original contributions presented in this study are included in the article. Further inquiries can be directed to the corresponding author.

## References

[1] Roebuck B., Prakash L. (2012). Innovations in Tungsten Carbide-Cobalt Hardmetal Technology. International Powder Metallurgy Directory 2012–2013.

[2] Dabees S., Mirzaei S., Kaspar P., Holcman V., Sobola D. (2022). Characterization and Evaluation of Engineered Coating Techniques for Different Cutting Tools—Review. Materials.

[3] Tkadletz M., Schalk N., Daniel R., Keckes J., Czettl C., Mitterer C. (2016). Advanced characterization methods for wear resistant hard coatings: A review on recent progress. Surf. Coat. Technol..

[4] PalDey S., Deevi S.C. (2003). Single layer and multilayer wear resistant coatings of (Ti, Al) N: A review. Mater. Sci. Eng. A.

[5] Vereshchaka A.S., Mgaloblishvili O., Morgan M.N., Batako A.D. (2014). Nano-scale multilayered-composite coatings for the cutting tools. Int. J. Adv. Manuf. Technol..

[6] Martinho R.P., Silva F.J.G., Martins C., Lopes H. (2019). Comparative study of PVD and CVD cutting tools performance in milling of duplex stainless steel. Int. J. Adv. Manuf. Technol..

[7] Yongqiang W., Xiaoya Z., Zhiqiang J., Xiubo T. (2018). Characterization and mechanical properties of TiN/TiAlN multilayer coatings with different modulation periods. Int. J. Adv. Manuf. Technol..

[8] Straumal B.B., Konyashin I. (2023). Faceting/Roughening of WC/Binder Interfaces in Cemented Carbides: A Review. Materials.

[9] Kupczyk M.J. (2014). Cutting edges with high hardness made of nanocrystalline cemented carbides. Int. J. Refract. Met. Hard Mater..

[10] Liu W., Chu Q., Zeng J., He R., Wu H., Wu Z., Wu S. (2017). PVD-CrAlN and TiAlN coated Si3N4 ceramic cutting tools—1. Microstructure, turning performance and wear mechanism. Ceram. Int..

[11] Ding X.Z., Samani M.K., Chen G. (2010). Thermal conductivity of PVD TiAlN films using pulsed photothermal reflectance technique. Appl. Phys. A.

[12] Zhao J., Liu Z., Shen Q., Wang B., Wang Q. (2018). Investigation of Cutting Temperature during Turning Inconel 718 with (Ti,Al)N PVD Coated Cemented Carbide Tools. Materials.

[13] Falsafein M., Ashrafizadeh F., Kheirandish A. (2018). Influence of thickness on adhesion of nanostructured multilayer CrN/CrAlN coatings to stainless steel substrate. Surf. Interfaces.

[14] Zhang K., Deng J., Guo X., Sun L., Lei S. (2018). Study on the adhesion and tribological behavior of PVD TiAlN coatings with a multi-scale textured substrate surface. Int. J. Refract. Met. Hard Mater..

[15] Palanisamy S., Rashid R.R., Brandt M., Sun S., Dargusch M. (2014). Comparison of endmill tool coating performance during machining of Ti6Al4V Alloy. Adv. Mater. Res..

[16] Palanisamy S., Rashid R.R., Brandt M., Dargusch M.S. (2014). Tool life study of coated/uncoated carbide inserts during turning of Ti6Al4V. Adv. Mater. Res..

[17] Rodríguez-Barrero S., Fernández-Larrinoa J., Azkona I., de Lacalle L.N.L., Polvorosa R. (2016). Enhanced performance of nanostructured coatings for drilling by droplet elimination. Mater. Manuf. Process..

[18] Pytlak B. (2015). Optimization of tool wear during plunge turning of hardened 18CrMo4 steel. Adv. Manuf. Sci. Technol..

[19] Beake B., Ning L., Gey C., Veldhuis S., Kornberg A., Weaver A., Khanna M., Fox-Rabinovich G. (2015). Wear performance of different PVD coatings during hard wet end milling of H13 tool steel. Surf. Coat. Technol..

[20] Iqbal A., Zhao G., Cheok Q., He N., Nauman M.M. (2022). Sustainable machining: Tool life criterion based on work surface quality. Processes.

[21] Wojciechowski S., Talar R., Zawadzki P., Legutko S., Maruda R., Prakash C. (2020). Study on Technological Effects of a Precise Grooving of AlSi13MgCuNi Alloy with a Novel WCCo/PCD (DDCC) Inserts. Materials.

[22] Chinchanikar S., Choudhury S. (2013). Wear behaviors of single-layer and multi-layer coated carbide inserts in high speed machining of hardened AISI 4340 steel. J. Mech. Sci. Technol..

[23] Liew W.Y., Jie J.L.L., Yan L.Y., Dayou J., Sipaut C., Bin Madlan M.F. (2013). Frictional and wear behaviour of AlCrN, TiN, TiAlN single-layer coatings, and TiAlN/AlCrN, AlN/TiN nano-multilayer coatings in dry sliding. Procedia Eng..

[24] Wu J., Zhan G., He L., Zou Z., Zhou T., Du F. (2020). Tribological Performance of Micro-Groove Tools of Improving Tool Wear Resistance in Turning AISI 304 Process. Materials.

[25] Ananthakumar R., Subramanian B., Kobayashi A., Jayachandran M. (2012). Electrochemical corrosion and materials properties of reactively sputtered TiN/TiAlN multilayer coatings. Ceram. Int..

[26] Rezaee S., Arman A., Jurečka S., Korpi A.G., Mwema F., Luna C., Sobola D., Kulesza S., Shakoury R., Bramowicz M. (2020). Effect of annealing on the micromorphology and corrosion properties of Ti/SS thin films. Superlattices Microstruct..

[27] Liu L., Jiang X., Yingam E., Sun Z., Geng D., Zhang D. (2025). High-performance milling of Ti-6Al-4V through rotary ultrasonic elliptical milling with anticlockwise elliptical vibration. J. Zhejiang Univ. Sci. A.

[28] Upadhyaya G.S. (1998). Cemented Tungsten Carbides. Production, Properties, and Testing.

[29] Oberg E., Jones F.D., Horton H.L., Ryffell H.H. (2000). Cemented Carbides. Machinery’s Handbook.

[30] Niihara K., Morena R., Hasselman D.P.H. (1982). Evaluation of *K*_Ic_ of brittle solids by the indentation method with low crack-to-indent ratios. J. Mater. Sci. Lett..

[31] (2007). Advanced Technical Ceramics. Mechanical Properties of Monolithic Ceramics at Room Temperature. Part 4. Surface Hardness According to: Vickers, Knoop and Rockwell.

[32] Dickson G. (1969). Ultrasonic Methods for Determination of Mechanical Properties of Dental Materials. National Bureau of Standards.

[33] (2022). Fine Ceramics (Advanced Ceramics, Advanced Technical Ceramics)—Determination of Density and Apparent Porosity.

[34] Vander Voort G.F. (1999). Metallography. Principles and Practice.

[35] (2016). Hardmetals—Metallographic Determination of Microstructure. Part 4: Characterisation of Porosity, Carbon Defects and Eta-Phase Content.

[36] (1993). Tool-Life Testing with Single-Point Turning Tools.

[37] Frydrych H., Konstanty J., Ratuszek W. (2006). Microstructure and properties of sintered cobalt-chromium carbide materials. Arch. Metall. Mater..

[38] Polcomm® DEGroove Reliable Polcomm Solutions for Parting & Grooving. https://www.polcomm.com.pl/wp-content/uploads/2023/04/Polcomm_DEGroove_Rowkowanie_Grooving.pdf.

